# Modern Sociology

**Published:** 1903-11-28

**Authors:** 


### Modern Sociology.

### THE CHILDREN'S COMMITTEE OF THE METROPOLITAN ASYLUMS BOARD.

This committee has sent out its fifth annual report, describing the work done during last year. Much of this, especially the work among the defective children, is a continuation of that of the previous year, but new work has been developed in the care of children suffering from ringworm and ophthalmia. At the end of last year neither of the proposed schools for the reception of ophthalmia children were open, but the lines of administration had been laid down. The schools are to be in the form of cottage groups, each group accommodating 72 children and nine officers. There will be four buildings in each group, *i.e.*, three pairs of cottages, each semi-detached cottage being occupied by a house-mother and 12 children, or 24 children and two house-mothers in each pair, while the fourth building contains a kitchen and scullery, store-rooms, rooms for the medical treatment of the children, and the rooms for the charge nurse. The relations of the charge nurse and the house-mother were a subject of considerable discussion among the members of the committee, but it was finally decided that so far as the ordinary life of the children was concerned, the house-mothers should be responsible only to the matron. It had been suggested that the house-mothers should be responsible to the charge nurse, but it was realised that the duties of the two were distinct, and that to increase the number of mistresses over the house-mothers would probably lead to friction. The house-mothers are plain, kindly women, prepared to do all household work, with such assistance as the children under their charge can give, and it was felt that this plan was better adapted to bring up the children in a way that would fit them for their future work than to put them in the charge of women trained as nurses, who might be unwilling to fulfil the ordinary duties of the home, and in matters of nursing might be less amenable to the control of the charge nurse. The arrangement by which there is one charge nurse to supervise the health-care of the children, while leaving the domestic training to the house-mother, is certainly that which best simulates the conditions of ordinary life, and is therefore in all probability the most wholesome for the children themselves. The next report of the committee will be interesting if it gives an account of the working of the schools.

The treatment of children suffering from ringworm was begun at the Bridge School, Witham, and a new school was to be opened at Banstead during the past summer. The Banstead school, which was taken over from the South Metropolitan School District Board, consisted, under that authority, of six self-contained blocks, each of which included a kitchen and a small hand laundry, besides the usual administrative block, and an infirmary at the porter's lodge. Each block contained accommodation for 70 ringworm children. Under the South Metropolitan authority the school had been used for girls only, and the elder girls had been able to give considerable help in the house-work, being in this way trained for domestic service. Among other things, they did the laundry work of their respective blocks under the supervision of one woman. The Committee of the Metropolitan Asylums Board, who now took over the school, resolved to administer it in the main on the same lines—each block under the charge of its own house-matron, but as many of the children would now be too young to give much help in the work, it was resolved to relieve them at least of the laundry work. Taking into consideration the inconvenience and expense of hiring outside washerwomen, and the undesirability of bringing in such women daily, it was resolved to build a laundry for the use of the schools. The existing building was some twenty years old, and it was found necessary to spend a good deal of money in bringing it up to date. Modern bathing, washing, and sanitary appliances were required, besides a great deal of painting and cleaning, and the total expense was expected to amount to £5,000. The work of medical supervision is of an exacting nature, as it involves an examination of the scalp of each child before signing a certificate for its discharge, and the committee note with satisfaction that, thanks to the care exercised before discharging any of the inmates, there have been only six readmissions.

It is rather strange to find that the only complaint which the committee have to make against the guardians of the various unions whose children they receive, is that these do not make as much use as they might of the seaside homes at Margate and Herne Bay. The complaint has been made before, but there has been no increase in the demand for accommodation at the homes. The committee say:—"We do not doubt that a periodical examination on the part of the officers of the guardians of the children under their care would reveal more than sufficient candidates needing the benefit of seaside air to fill the homes." The homes being there it certainly seems a pity that the children should not get the benefit of them. It is desired to erect a verandah at the home at Margate (East Cliff House). Many of the children sent there are suffering from tubercular diseases, especially tubercular disease of joints and spine, tubercular glands, and early cases of phthisis. For them the open-air treatment would be very beneficial, and if the cases were treated early enough many might be cured. A verandah where they could lie out all day long would facilitate this, and render them comparatively independent of the weather, whereas at present many of them can take the air only in a donkey cart or in a spinal chair. A new seaside house is in process of erection at Millfield, Rustington.

The homes for defective children continue to do useful work, though in all, and especially in the schools for the feeble-minded, the character of the children makes it difficult to report marked progress. It has been found possible to send a feeble-minded girl from the home at Lloyd Street into service at the Soho Home for Working Girls, and the report from there is that her domestic work is good, but her character is difficult. At the Home at Kingwood Road, one or two new features have been started. One is the allowing the bigger boys to go out for walks unaccompanied, and another the allowing of a penny a week pocket-money, which may be wholly or partially stopped for misbehaviour, and the possession of which will, it is hoped, teach those who receive it something about the care and value of money.

The committee plead for an extension of the privileges of the remand homes. They say:—"It would appear of little advantage to make elaborate provision for preventing the young, after their first appearance before a magistrate, from passing a week or two in a workhouse, when the first night after the arrest is spent in a police cell adjacent to hardened criminals." "There seems to be no valid reason why some means should not be devised for extending the usefulness of these homes in the direction of receiving the children directly on arrest at the Homes, and of appointing a special magistrate to deal with juvenile offenders, and one who could very possibly attend at the Homes to hear the complaints." This would certainly carry out much better than the existing method the intention of the Homes—the separation of youthful offenders from hardened criminals.

